# Isolation and Characterisation of Plant Growth-Promoting Rhizobacteria for Improving Growth and Water/Salt Stress Resilience in Lettuce

**DOI:** 10.3390/microorganisms14020353

**Published:** 2026-02-03

**Authors:** Diana Di Gioia, Francesca Gaggìa, Marco Bosco, Elia Pagliarini, Loredana Baffoni

**Affiliations:** Department of Agricultural and Food Sciences, Alma Mater Studiorum-Università di Bologna, Viale Fanin 42, 4012 Bologna, Italy; diana.digioia@unibo.it (D.D.G.); francesca.gaggia@unibo.it (F.G.); marco.bosco@unibo.it (M.B.); loredana.baffoni@unibo.it (L.B.)

**Keywords:** PGPR, *Lactuca sativa* L., salt stress, drought stress, greenhouse

## Abstract

Plant Growth-Promoting Rhizobacteria (PGPR), represent a promising tool for the development of sustainable agriculture practices. Although numerous strains have been described in the literature, their characterisation often overlooks the ability to sustain functional activity under common abiotic stress conditions, such as water deficit and high salinity. The present study aimed to isolate putative PGPR strains from different environmental and biological matrices, characterise their key plant growth-promoting traits, and evaluate their effectiveness in improving plant growth under water and salt stress conditions. The isolated strains were initially tested in vitro for phytohormone production, phosphate solubilisation, and siderophore production. Selected *Bacillus* and *Pseudomonas* strains exhibiting the most promising traits were tested in a preliminary greenhouse pot test using lettuce (*Lactuca sativa*), followed by assays under drought stress (50% water reduction) and salt stress (100 mM NaCl). The results demonstrated that the two *Bacillus velezensis* strains (PB_8 and CSS_12) significantly enhanced plant growth by increasing foliar biomass and root development improving pigment content, and mitigating stress-induced damage. Overall, these findings support the potential of PGPR-based strategies for low-impact agricultural practices and enhancing plant resilience under stress conditions.

## 1. Introduction

Plant Growth-Promoting Rhizobacteria (PGPR) provide a sustainable alternative to chemical fertilisers and pesticides, offering an effective strategy to reduce the environmental impact of agricultural practices. PGPR are a diverse group of soil bacteria that colonise the rhizosphere [1,2] and interact beneficially with plants, enhancing plant growth, productivity and resilience to environmental stresses [3,4]. PGPR alleviate plant stress through both direct and indirect mechanisms. Direct mechanisms include increased nutrient availability (nitrogen fixation, phosphorus solubilisation), water uptake, exopolysaccharide production, biofilm formation, and the secretion of organic solutes such as sugars, organic acids, amino acids, and polyamines. PGPR reduce plant stress induced by heavy metals through indirect mechanisms such as siderophore production. These siderophores chelate iron in the rhizosphere, improving its availability for plants, for chlorophyll synthesis and photosynthetic efficiency. Additionally, siderophores chelate heavy metals including cadmium, lead, and zinc, reducing their bioavailability and toxicity to plants [5]. Indirect mechanisms also include phytohormones production, such as indole-3-acetic acid (IAA), gibberellins, and auxins, which stimulate root growth, improving nutrient and water uptake. The hormonal signalling mediated by rhizobacteria plays a key role in regulating plant responses to biotic stressors, with a possible induction of systemic resistance to pathogens [6,7]. Several PGPR strains can produce antimicrobial substances that directly inhibit the growth of harmful microorganisms in soil or degrade ethylene precursors reducing ethylene production in stressed plants [8,9]. PGPR have potential as alternatives to chemical fertilisers and pesticides. One problem is that their performance in field conditions is highly variable, due to environmental factors and different soil composition. Many PGPR strains selected in controlled conditions do not perform well under real stress conditions, and this limits their use. Moreover, although the beneficial effects of PGPR have been widely investigated in several crop species, including lettuce, most studies have focused on single stress conditions or specific growth stages, often under controlled experimental environments [10,11].

Intensive anthropogenic activities, combined with climate change, have led to a systematic decline in soil fertility and plant biomass production [12]. Consequently, plants growing under field conditions face a multitude of environmental challenges, such as drought, salinity, high temperature, and nutrient deficiency, that often occur simultaneously, compounding stress severity. These abiotic stresses can adversely affect plant growth and health by disrupting physiological and biochemical processes required for survival and yield [13]. Beneficial microorganisms can improve plant tolerance to environmental stressors by modulating stress-responsive pathways and maintaining plant physiological homeostasis [12,14,15]. However, the combined effects of multiple abiotic stresses and the role of PGPR in modulating plant responses under such complex stress scenarios remain insufficiently explored, particularly in leafy vegetable crops. Among abiotic stress factors, salinity is one of the most critical constraints affecting plant growth and productivity. Soil salinisation is closely associated with climate change and agricultural intensification, leading to significant reductions in crop yields [16,17]. This is especially prevalent in areas characterised by saline soil or irrigation with saline waters. High salt concentration in the soil environment impedes water absorption, disrupts the nutrient uptake process [18], and can cause oxidative damage [19], thereby reducing the quantity and quality of crops produced [20,21,22,23]. Water availability is another crucial factor limiting the growth and productivity of many crops, including lettuce (*Lactuca sativa* L.). This species is particularly sensitive to water availability, which underlines its vulnerability to water stress [24]. Water is essential for maintaining cell turgidity and expansion; under limited moisture conditions, lettuce leaves grow and leaf and root biomass are reduced. This leads to the inhibition of plant growth and reduction in total biomass [25]. Drought stress directly affects the size and quality of lettuce heads by slowing cell division. As water availability declines, lettuce plants exhibit leaf wilting and curling, stomatal closure, and reduced transpiration, resulting in decreased photosynthetic activity [26]. Additionally, water limitation reduces the uptake of essential nutrients, particularly those transported via mass flow, such as nitrogen and potassium [27]. Despite the agronomic relevance of salinity and water deficits for lettuce cultivation, studies addressing both effects on plants and the potential of PGPR to alleviate these stressors remain limited [28].

The aim of this work was to investigate the potential role of newly isolated PGPR strains in mitigating salt and water stresses on lettuce growth and development. Particularly, the selected bacterial species were studied for their capability of surviving and exerting their beneficial activity in harsh environments, conferring resilience to plants under both high salinity and limited water availability stress.

## 2. Material and Methods

### 2.1. Isolation and Molecular Identification

Bacterial isolation was performed by taking 1 g of the different matrices (vegetable tissues, chicken manure and coffee silver skin, described in more detail in Section 2.2), and suspending in 99 mL of saline solution (NaCl 0.9% *wt*/*v*) before homogenizing using a Stomacher lab blender (Colworth STOMACHER 400, Seward, England) for 30 min. Bacteria were isolated by the pour plate method after serial dilutions of the homogenised solution and plating on count agar (TSA; Oxoid Milan, Italy). Plates were incubated at 37 °C for 24 h under aerobic conditions to selectively recover fast-growing and stress-tolerant microorganisms, with potential applicability in agricultural and biotechnological contexts. Although this condition is known to favour the growth of *Bacillus* species, it does not preclude the isolation of other PGPR genera, as confirmed by the recovery of other bacterial strains in the present study. This approach was adopted in order to select microorganisms that are more likely to maintain their functionality under abiotic stress conditions. This temperature falls within the optimal growth range of *Bacillus* strains, which are frequently isolated as PGPR, and is consistent with the ecological origin of some studied matrices, such as organic material and heat-treated food byproducts. Each analysis was carried out in duplicate. The isolated colonies were enumerated, and the number of bacteria was calculated as Log of Colony Forming Unit (CFU)/g. Isolates were re-streaked on the same medium to ensure strain purity. Pure cultures were stored in 20% glycerol solution at −80 °C. The isolates were identified by sequencing the 16S rRNA gene. Genomic DNA was extracted using the Wizard^®^ Genomic DNA Purification Kit (Promega, Madison, WI, USA). The 16S rRNA gene was amplified using the universal bacterial primers 8F (5′-AGA GTT TGA TCC TGG CTC AG-3′) and 1520R (5′-AAG GGA GGT GAT CCA GCC GCA-3′) under the following conditions: denaturation at 95 °C for 2 min, followed by 35 cycles of 95 °C for 15 s, 55 °C for 1 min, 72 °C for 1 min, and final extension at 72 °C for 10 min. Amplified PCR results were analysed by electrophoresis using 1.5% agarose gel supplemented with SyberSafe (Invitrogen, Waltham, MA, USA) and photographed using the gel documentation GelDoc™ System (Bio-Rad, Milano, Italy). The purification of the PCR products was carried out using the NucleoSpin purification kit (Macherey-Nagel GmbH & Co. KG, Düren, Germany), and the subsequent sequencing was performed by Eurofins MWG Operon (Ebersberg, Germany). The software program Finch TV version 1.4 was used to edit the sequence chromatograms, and the consensus sequence was built using DNAMAN software version 6.0 (Lynnon BioSoft, Inc., San Ramon, CA, USA). Sequence assignment to species or genera was investigated by matching available 16S rRNA sequences using the Basic Local Alignment Search Tool (BLAST; http://www.ncbi.nlm.nih.gov/BLAST/) accessed on 8 June 2021 [29].

### 2.2. Matrices of Bacterial Isolation

Microbial isolates were obtained from a range of environmental and biological matrices, which were selected to represent different ecological niches and exposures to environmental or technological stresses. Vegetable-associated matrices included rhizosphere samples and plant tissues collected from potted *Zingiber officinale* plants grown under water stress conditions. These matrices were selected to enhance the probability of isolating microorganisms adapted to drought-related stress and capable of interacting closely with plant root systems. *Pseudomonas taiwanensis* T_2_C and *Pseudomonas plecoglossicida* T_1_R were isolated from these samples. Fruit-associated matrices were represented by peach (*Prunus persica*) fruits, which were collected from plants grown in agricultural soil under an intensive cultivation system that was irrigated with secondary-treated wastewater. This environment is characterised by elevated microbial pressure and variable abiotic conditions, which may favour the selection of stress-tolerant, potentially endophytic microorganisms. *Bacillus velezensis* PB_8 and *Bacillus tropicus* PB_7 were isolated from these samples. Additional rhizosphere samples were collected from oak (*Quercus robur*) trees growing in an agroforestry system in the hilly Emilia-Romagna region of Italy. This perennial woody system is characterised by complex and stable root exudation patterns that are distinct from those of annual herbaceous crops and which potentially harbour microbial communities with unique functional traits. *Bacillus toyonensis* QR_6 was isolated from these samples. *Bacillus halotolerans* CSS_12_6, *Bacillus mojavensis* CSS_C9, *Bacillus tequilensis* CSS_7_3 and *Bacillus velezensis* CSS_12_1 were isolated from coffee silver skin, a food waste product that undergoes a roasting process, which enables them to resist specific environmental stresses [30]. *Stenotrophomonas maltophilia* CM_4_4 was finally isolated from chicken litter manure collected from an intensive poultry farming system. This material is rich in organic matter and nutrients, and is usually subjected to thermal and microbial fermentation processes. These conditions may promote the selection of microorganisms that are tolerant of high temperatures and variable physicochemical conditions [29].

The final list of strains selected for in vitro tests and the references related to the first preliminary pot experiment are: *Pseudomonas taiwanensis* T_2_C (thesis reference: PT), *Pseudomonas plecoglossicida* T_1_R (thesis reference: PP), *Bacillus mojavensis* CSS_C9 (thesis reference: BM), *Stenotrophomonas maltophilia* CM_4_4 (thesis reference: SM), *Bacillus halotolerans* CSS_12_6 (thesis reference: BH), *Bacillus velezensis* PB_8 (thesis reference: BV1), *Bacillus toyonensis* QR_6 (thesis reference: BT1), *Bacillus tropicus* PB_7 (thesis reference: BT2), *Bacillus tequilensis* CSS_7_3 (thesis reference: BT3) and *Bacillus velezensis* CSS_12_1 (thesis reference: BV2).

### 2.3. PGPR Characterisation

#### 2.3.1. Phosphate Solubilisation

Phosphate solubilisation was assessed using Pikovskaya’s Agar plates [31]. Briefly, 50 µL of exponentially grown PGPR cultures were spot inoculated on plates, incubated at 28 °C for 7 days, and P-solubilising activities were analysed in triplicate. The formation of a halo around bacteria colonies confirmed the solubilisation of the phosphate: +, small halos < 0.5 cm; ++, medium halos > 0.5 cm; +++, large halos > 1.0 cm [32,33].

#### 2.3.2. Siderophore Production

Siderophore production was determined on Chrome-azurol S (CAS) medium following the method of Schwyn and Neilands [34]. The bacterial strains (24 h old cultures) were spotted separately on CAS medium. Plates were incubated at 30  ±  1 °C for 120 h. Formation of an orange to yellow halo around the colonies showed the production of siderophore [35].

#### 2.3.3. Indole-3-Acetic Acid (IAA) Production

Isolates were evaluated for their ability to synthesise IAA, thus stimulating plant growth. IAA production was determined by using Gordon and Weber’s approach [36]. IAA production in the medium leads to a red-pink colour formation. In brief, 10 mL of NB medium supplemented with 0.1% (*w*/*v*) L-tryptophan (Sigma-Aldrich^®^, Merk KGaA, Burlington, MA, USA) were inoculated with fresh bacterial cultures in the dark under the following condition: shaking for 72 h at 28 °C. Then, 1 mL of supernatant, obtained by centrifugation (5000× *g*/10 min at 4 °C), was mixed with 3 mL Salkowski’s reagent (2% 0.5 M FeCl_3_ in 35% HClO_4_ solution) and kept in the dark. After 30 min incubation, the amount of IAA was spectrophotometrically determined (540 nm). The IAA concentration was estimated using an IAA standard curve [37].

### 2.4. Preliminary Pot Experiment Design

The preliminary experiment was performed with all 10 strains listed above (Section 2.1) in April 2023 in a protected greenhouse at the University of Bologna (Bologna, Italy). The seedlings of homogeneous lettuce (*Lactuca sativa* L. cv. Gentile), with three leaves, were supplied by Orto Mio s.r.l. (Verona, Italy) 20 days after sowing and transplanted into individual pots (capacity 2 L). The pots were filled completely with the same substrate composed of acid peat, wet peat and uncomposted green soil conditioner (pH 7.5; total porosity *v*/*v*: 80%; dry bulk density 450 kg/m^2^; supplied by Gramoflor GmbH & Co., Vechta, Germany). The temperature was controlled within the range of 15 °C (minimum) and 33 °C (maximum). The vessels were arranged in completely randomised blocks, with 10 different microbial treatments and the control. Microorganisms were inoculated at doses of 10^9^ CFU/mL every 10 days until sample collection. Twelve pots were used for each microbial treatment. A harvest cycle of 33 days from transplantation to harvest was used.

### 2.5. Second Pot Experiment Design

This experiment was conducted in a protected greenhouse at the University of Bologna (Bologna, Italy) in April 2024. The lettuce seedlings (20 days; variety *Lactuca sativa* cv. Gentile) were provided by Orto Mio s.r.l. (Verona, Italy), transplanted into 2 L pots with the substrate used previously and cultivated for 33 days. For this experiment (Table 1), a selection of previously tested strains was used and applied in three cultivation conditions: normal conditions, salt stress (S) and drought stress (D). For each condition there was an untreated control (without microorganisms) and various treatments involving the application of microorganisms at the concentration of 10^9^ CFU/mL. Plants were irrigated every 2 days with tap water under normal conditions, and with water containing 100 mM NaCl under salt stress conditions. Meanwhile, the plants with drought stress were subjected to a 50% reduction in water. Each treatment consisted of 12 pots for the respective conditions.

### 2.6. Inoculum Setup

Microbial strains were grown in 50 mL Tryptone Soy Broth (TSB, Oxoid, ThermoFisher, Waltham, MA, USA) for 24 h at 30 °C with horizontal shaking. The microbial biomass was then inoculated into 1 L of TSB and grown at 30 °C until they reached 2.0 × 10^9^ colony forming units (CFU ∙ 10 mL^−1^). Bacteria were collected by centrifugation at 5000× *g* for 10 min and then resuspended in 100 mL of sterile deionised water. Treatments were carried out by watering, with 10 mL of water added to the bottom of each plant to achieve a final quantity of 10^9^ CFU in each pot. This amount is in agreement with a previous work, in which different amount of inoculum were considered [38]. An equal volume of tap water was added to control plants, but no other nutrients or microbial inoculants were used.

### 2.7. Sampling and Yield Assessment

#### 2.7.1. Preliminary Pot Experiment

Thirty-three days after transplantation (10 days after the last inoculum), nine plants from each treatment and control were randomly selected and transferred to the laboratory. Plants were cut at the collar within 3 h; fresh weight (FW) was determined by weighing lettuce heads immediately. The above-ground material was then placed in a paper bag and dried at 70 °C for 72 h. The difference in weight before and after drying was used to calculate sample shoot dry weight (DW). Dry matter percentage (DM%) was calculated as DW/FW×100.

#### 2.7.2. Second Pot Experiment

Nine plants per treatment were randomly selected and transported to the laboratory in April 2024, 33 days after transplanting and 10 days after the last treatment. The determination of FW, DW and DM % was carried out as described in the previous section. Measurements of the fresh weight (FW) and dry weight (DW) of the roots were also carried out, as well as the corresponding calculations of the dry matter percentage (DM%).

#### 2.7.3. Chlorophylls and Carotenoids Determination

For the determination of chlorophyll a, chlorophyll b, and carotenoids, leaf samples of three plants were ground in acetone and centrifuged without drying. The absorbance of the supernatant was measured at 665 nm, 649 nm and 470 nm according to the methods of Porra et al. [39] and Lichtenthaler et al. [40].

### 2.8. Statistical Analyses

Experimental data from leaves and roots (FW, DW and DM%), as well as chlorophylls, carotenoids, IAA production, were analysed separately using one-way ANOVA and HSD Tukey’s post hoc analysis at *p* < 0.05, which was performed using R software (v 4.3.3) [41].

## 3. Result and Discussion

### 3.1. Isolation

The following microorganisms were isolated from different matrices and are shown in Table 2. It should be acknowledged that the isolation strategy adopted in this study, which included incubation at 37 °C, may have influenced the taxonomic composition of the recovered PGPR by favouring stress-tolerant genera such as *Bacillus*. However, this methodological choice was in line with the objective of identifying microorganisms capable of surviving and exerting beneficial effects in adverse environments. The inclusion of non-spore-forming bacteria, such as *Pseudomonas*, among the selected strains indicates that the study focused on functional resilience and stress tolerance as key selection criteria, rather than being restricted to *Bacillus*-based PGPR. Overall, all strains were selected based on their isolation from matrices exposed to different environmental and technological stresses, suggesting an intrinsic ability to adapt to variable and challenging conditions.

### 3.2. PGPR Characterisation

Regarding the results of the PGPR traits evaluation, all the tested strains formed transparent halos around the spot inoculation, although with different solubilizing activity. Only strain BT1 did not possess this activity (Table 3). The most performant strain in phosphate solubilisation was PT, and BT2, BV1, and BV2 also showed good capacity. Several studies have reported a high capacity for solubilizing inorganic and organic P by bacterial activity, mainly by *Bacillus* species [42]. The ability to solubilise phosphorus is of great importance for enhancing soil fertility, particularly in phosphorus-deficient soils.

All tested strains were found to produce siderophores, apart from *B. toyonensis* QR_6 (Table 3). Similar results have been observed in other studies, where *Bacillus* and *Pseudomonas* species demonstrated good siderophore production capabilities [35,43]. Significant variability in IAA production among the strains was observed (Table 3). After 24 h, BT3 produced the highest levels of IAA, with further increased production after 72 h. In contrast, BT1 consistently showed the lowest IAA production across all three time points.

IAA is well-known for its role in regulating plant growth by stimulating root development. Previous studies have highlighted the ability of *Pseudomonas* and *Bacillus* species to increase root growth, biomass, and overall plant health through their IAA-producing activity [44,45]. Both *Bacillus velezensis* (CSS_12_1 and PB_8) have been reported to produce IAA in a dose-dependent manner, and this production is associated with increased root length and plant biomass under controlled conditions [46].

### 3.3. Preliminary Pot Experiment

This experiment was designed to evaluate the effects of bacterial inoculation on lettuce growth by comparing leaf and root biomass production with the in vitro characterisation of the strains, with the aim of identifying the most effective strain. The effects of microbial inoculum on lettuce plants were assessed in a preliminary greenhouse experiment by measuring fresh and dry biomass (FW and DW) of leaves (Table 4) and roots, as well as the percentage of total dry matter (DM%) and the photosynthetic pigment content (Table 5). Significant differences in the structural plant were observed in the FW values in the root development (Table 6). Particularly, PT, BM, BT2, BT3, and BV2 demonstrated significantly higher levels in the roots biomass compared to the untreated control. However, significant differences were observed in photosynthetic pigment content (Table 5). The highest values were recorded in the PT treatment, followed by the BM and BH treatments, which outperformed the untreated control.

The in vitro screening tests showed marked differences in PGPR traits (Table 3), with some strains showing the overall highest value of phosphate solubilisation, siderophore and indol-3-acetic acid production. Whereas the foliar biomass production did not show marked changes upon microbial inoculation (Table 4), differences among strains were clearly confirmed by root development parameters, which were in line with the highest values of PGPR traits. Therefore, PGPR characteristics and root biomass production were the most important parameters in our selection. Additionally, we checked that the strains showing the best-performing PGPR traits and the highest root biomass production produced a high level of photosynthetic pigments. The increase in carotenoid content observed in bacteria-treated plants may represent a significant qualitative improvement in lettuce biomass, which partially compensates for the lack of significant effects on above-ground growth. This is particularly relevant for leafy vegetables, where nutritional quality is a key characteristic. Although some strains, in particular *B. halotolerans* strain CSS_12_6, showed high levels of photosynthetic pigments in the leaves, we preferred not to select this strain for the subsequent test because of the low increase in roots biomass.

### 3.4. Second Pot Experiment

In the second greenhouse experiment, the best-performing bacterial strains were chosen based on the results of the in vitro screening and the previous greenhouse trial. The second greenhouse experiment was conducted to assess the ability of selected PGPR strains to enhance lettuce growth under abiotic stress conditions. Plants were grown under three cultivation regimes: optimal conditions, salt stress (100 mM NaCl), and drought stress (50% reduced irrigation), each including an untreated control.

**Figure 1:**
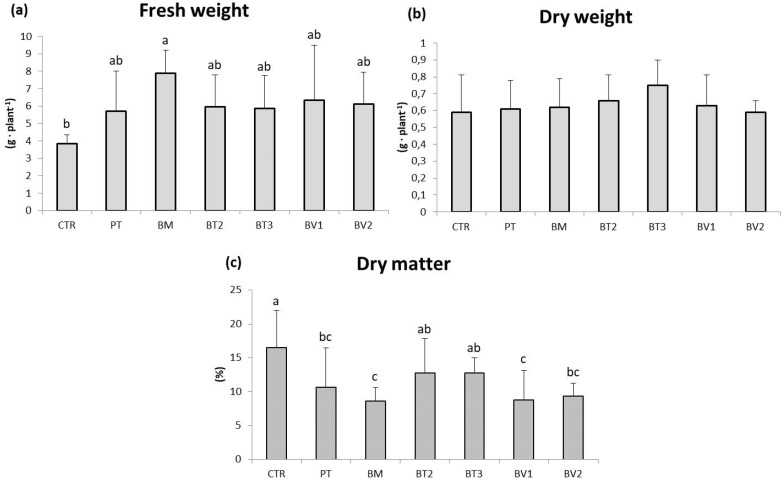
Roots fresh weight (**a**), dry weight (**b**), and dry matter (**c**) in plants in the second pot experiment without abiotic stresses. Mean values (±SD); (^a, b, c^) different letters in each graphs represent statistically significant differences based on Tukey’s post hoc test (*p* ≤ 0.05), no letters indicate no significant effect. CTR: control thesis, PT: *P. taiwanensis* T_2_C, BM: *B. mojavensis* CSS_C9, BT2: *B. tropicus* PB_7, BT3: *B. tequilensis* CSS_7_3, BV1: *B. velezensis* PB_8, and BV2: *B. velezensis* CSS_12_1.

**Figure 2:**
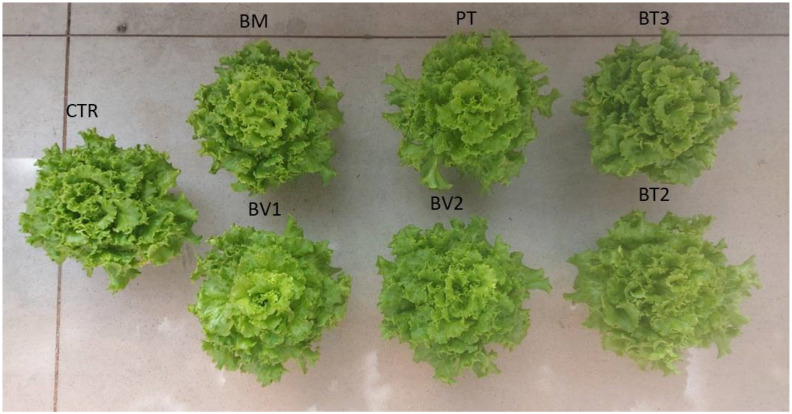
Second pot experiment with normal condition; CTR: control thesis, BM: *B. mojavensis* CSS_C9, PT: *P. taiwanensis* T_2_C, BT3: *B. tequilensis* CSS_7_3, BV1: *B. velezensis* PB_8, and BV2: *B. velezensis* CSS_12_1, BT2: *B. tropicus* PB_7.

BM-treated plants showed a significantly higher FW value compared to the control (Table 7). Similarly, photosynthetic pigment levels were higher in microbial-treated plants compared to the untreated control. Finally, carotenoid content was significantly higher in BT2-treated plants, followed by BV2 and BV1, with values approximately twice those of the control (Table 7). In addition, the marked enhancement of carotenoid and chlorophyll content indicates higher photosynthetic pigment content and improved nutritional quality. These pigments, beside optimising light harvesting for carbon fixation, provide antioxidant protection against photo-oxidative damage, which enhances the nutraceutical value of leafy biomass [47]. Specifically, Figure 1 shows that the BM-treated tissue exhibited significantly higher values of FW in roots compared to the control. As shown in Figure 2, preliminary test results revealed some differences in development under normal growth conditions.

These results confirm that, although not with significant increases, microbial inoculation can contribute to leaves growth by influencing roots development and promoting greater nutrient uptake. This is reflected in the increased photosynthetic pigment content, which improves the nutritional quality of the plants.

#### 3.4.1. Salt Stress

Analysis of fresh leaf biomass revealed that the BV-treated plants showed statistically the highest FW production compared to the control (Table 8).

Although the other bacterial strains did not significantly affect leaf biomass, differences emerged in the analysis of photosynthetic pigments. Both BT2 and the control (CTR) exhibited lower chlorophyll a content. In contrast, significantly higher values were recorded in PT- and BT3-treated plants. A similar trend was observed for chlorophyll b and carotenoid content. The highest values were found in BT3- and PT-treated plants (Table 8).

**Figure 3:**
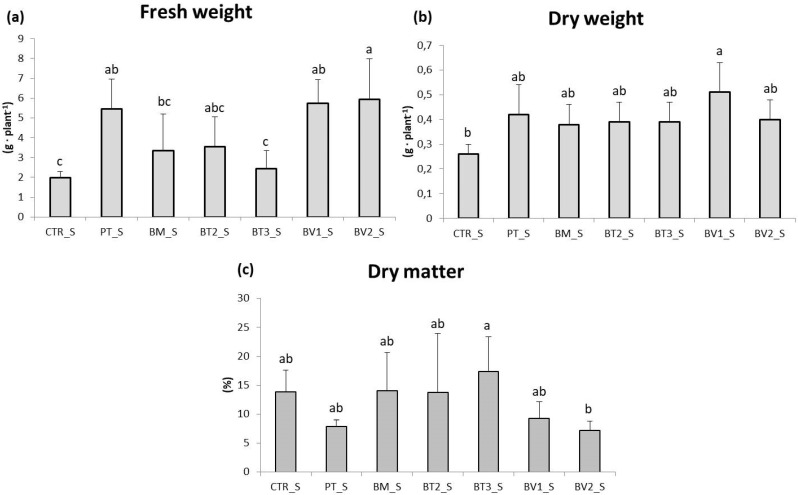
Roots fresh weight (**a**), dry weight (**b**), and dry matter (**c**) in plants in the second pot experiment with salt stress. Mean values (±SD); (^a, b, c^) different letters in each graphs represent statistically significant differences based on Tukey’s post hoc test (*p* ≤ 0.05). CTR_S: control thesis, PT_S: *P. taiwanensis* T_2_C, BM_S: *B. mojavensis* CSS_C9, BT2_S: *B. tropicus* PB_7, BT3_S: *B. tequilensis* CSS_7_3, BV1_S: *B. velezensis* PB_8, and BV2_S: *B. velezensis* CSS_12_1.

**Figure 4:**
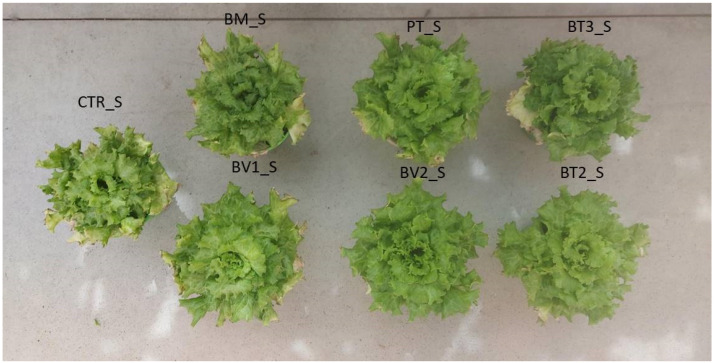
Second pot experiment with salt stress; CTR_S: control thesis, BM_S: *B. mojavensis* CSS_C9, PT_S: *P. taiwanensis* T_2_C, BT3_S: *B. tequilensis* CSS_7_3, BV1_S: *B. velezensis* PB_8, and BV2_S: *B. velezensis* CSS_12_1, BT2_S: *B. tropicus* PB_7.

In the roots (Figure 3), fresh weight was assessed, revealing significantly higher values in the BV2 treatment, BV1 treatment and PT treatment compared to CTR. These results indicate that, despite the continuous accumulation of NaCl due to irrigation, root development was more advanced in treated plants, likely due to enhanced nutrient uptake and improved soil exploration. This experiment demonstrated that salinity reduced leaf biomass and root length (Figure 4). Acosta-Motos et al. [48] suggested that growth retardation should be considered a key indicator when assessing salt stress.

In this study, the growth indices of lettuce plants decreased as salt concentration increased, with evident growth retardation, underscoring the plants’ sensitivity to salt stress. Previous studies also reported slowed growth and decreased biomass in plants exposed to saline soils [49,50].

Similarly, a study by Bai et al. [51] observed that *B. velezensis* mitigated the effects of salt stress on lettuce growth. PGPR inoculation was shown to support better growth and higher chlorophyll content under salt stress, suggesting that microorganisms enhance salt tolerance through multiple mechanisms. PGPR may improve plant resilience to salinity by enhancing nutrient uptake, producing phytohormones such as auxins and cytokinins, and reducing oxidative damage via antioxidant production. This involves enhancing nutrient uptake despite ion competition and producing phytohormones that antagonise stress-induced ethylene accumulation [52]. By promoting healthier root systems, PGPR likely enhanced the plant’s ability to absorb water and essential nutrients, mitigating the adverse effects of salinity on growth and chlorophyll content.

PGPR can help mitigate the impact of salt stress through several mechanisms. One of the main ways PGPR alleviates salt stress is by improving root development and nutrient acquisition. The production of PGPR, particularly those that produce exopolysaccharides (EPSs), has been linked to increased drought tolerance in plants, as these compounds can retain water in the rhizosphere by reducing osmotic stress [53] and improving soil structure, creating a more favourable environment for root growth during dry conditions [54,55]. In addition, PGPR can regulate plant hormonal balance, promoting root growth and improving overall plant development, even under stress conditions [56]. By enhancing root systems, PGPR-treated plants can more effectively absorb water and essential nutrients, which are often limited under saline conditions. In particular, high levels of IAA production stimulate root elongation and branching. This counteracts the inhibition of root growth typically induced by salinity-mediated ethylene accumulation [57]. Additionally, PGPR can induce the synthesis of antioxidants such as superoxide dismutase and peroxidase, which help mitigate oxidative stress caused by salt-induced reactive oxygen species (ROS). This likely explains why PGPR-treated plants retain higher chlorophyll content than uninoculated stressed plants, as chlorophyll degradation is typically associated with oxidative damage [58].

#### 3.4.2. Drought Stress

**Figure 5:**
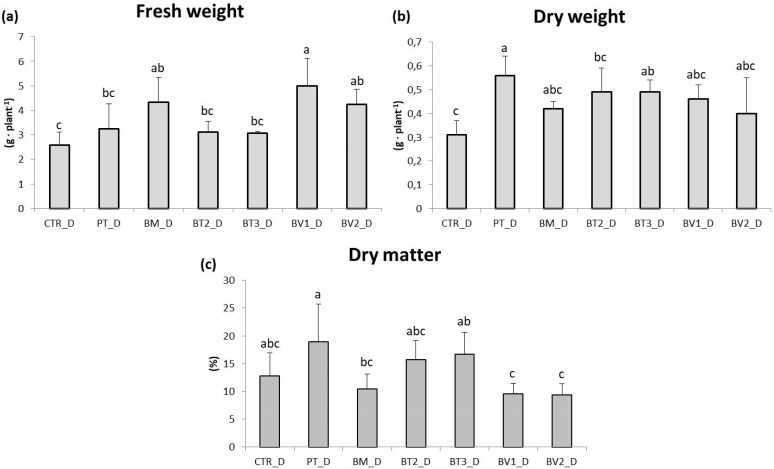
Roots fresh weight (**a**), dry weight (**b**), and dry matter (**c**) in plants in the second pot experiment with drought stress. Mean values (±SD); (^a, b, c^) different letters in each graphs represent statistically significant differences based on Tukey’s post hoc test (*p* ≤ 0.01). CTR_D: control thesis, PT_D: *P. taiwanensis* T_2_C, BM_D: *B. mojavensis* CSS_C9, BT2_D: *B. tropicus* PB_7, BT3_D: *B. tequilensis* CSS_7_3, BV1_D: *B. velezensis* PB_8, and BV2_D: *B. velezensis* CSS_12_1.

**Figure 6:**
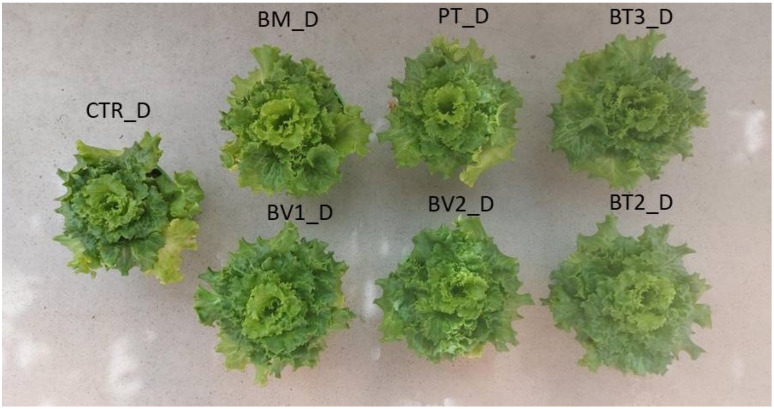
Second pot experiment with drought stress; CTR_D: control thesis, BM_D: *B. mojavensis* CSS_C9, PT_D: *P. taiwanensis* T_2_C, BT3_D: *B. tequilensis* CSS_7_3, BV1_D: *B. velezensis* PB_8, and BV2_D: *B. velezensis* CSS_12_1, BT2_D: *B. tropicus* PB_7.

The impact of water stress on lettuce (*Lactuca sativa*, cv. Gentile) growth was studied using different microbial treatments and an untreated control (Figure 5). Lettuce plants under water stress did not show a significant reduction in leaf area or fresh biomass compared to the control (*p* < 0.05). Moreover, microbial treatments did not increase leaf biomass compared to the control without microorganisms. Notably, the PT and BT2 treatments resulted in a significant reduction in fresh weight compared to the control, reflecting a “survival-over-growth” strategy where resource allocation prioritises cellular protection and photosynthetic apparatus integrity over biomass accumulation under limiting water availability [59]. Significant differences were observed in photosynthetic pigment content (Table 9). Chlorophyll a and chlorophyll b were highest in the BM treatment, followed by PT. Carotenoid content was highest in BT2 and BM. Particularly, BT2 exhibited significantly enhanced carotenoid accumulation, likely representing a critical photoprotective mechanism for scavenging ROS and protecting chlorophyll from photo-oxidative degradation under drought-induced stomatal closure [60]. Similarly, BM exhibited a higher amount of chlorophyll, suggesting improved photosynthetic performance, a key parameter for water-use efficiency under deficit irrigation [61]. The beneficial effects observed in microbe-treated plants under water stress could be interpreted as the result of physiological adjustments related to stomatal regulation and water-use efficiency. These adjustments are widely recognised as key components of microbial-mediated drought tolerance. This adaptive response is critical for maintaining plant hydration and reducing transpiration losses, a common issue under drought conditions [62]. Higher chlorophyll content in microbe-treated plants further supports this finding, indicating improved photosynthetic efficiency despite water deficit. While chlorophyll degradation is a typical response to water deficit, microbial treatments appeared to mitigate this effect, enabling plants to sustain photosynthetic activity under stress. Microbial treatments also had a significant effect on root development (Figure 5). The BM and BV1 treatments yielded root fresh biomass compared to the inoculated control. Under water stress, untreated lettuce plants showed significant reductions in root length and root biomass, with decreases of approximately 50%. Water stress typically limits root growth due to limited water availability, compromising the plant’s ability to absorb nutrients and moisture; Figure 6 shows plants subjected to drought stress. However, bacterial inoculations promoted root growth even under stress conditions, allowing plants to access deeper soil layers for water and nutrients [63]. The increased root biomass is in line with the in vitro IAA-producing capacity of these strains. Recent evidence shows that bacterial indole-3-acetic acid stimulates the growth of secondary roots by modulating cell wall loosening and increasing the surface area of the root. This allows the roots to explore deeper soil horizons for residual moisture [64]. The increase in root biomass and length observed in plants treated with microorganisms positively influenced overall root development. Beneficial bacteria stimulate root growth by producing plant hormones such as auxins, which promote root elongation, branching, and overall root health [56]. Additionally, some bacterial species improve soil structure and nutrient availability, enhancing root development and water uptake under water stress conditions [65].

#### 3.4.3. Strains Performance Under Different Conditions

**Figure 7:**
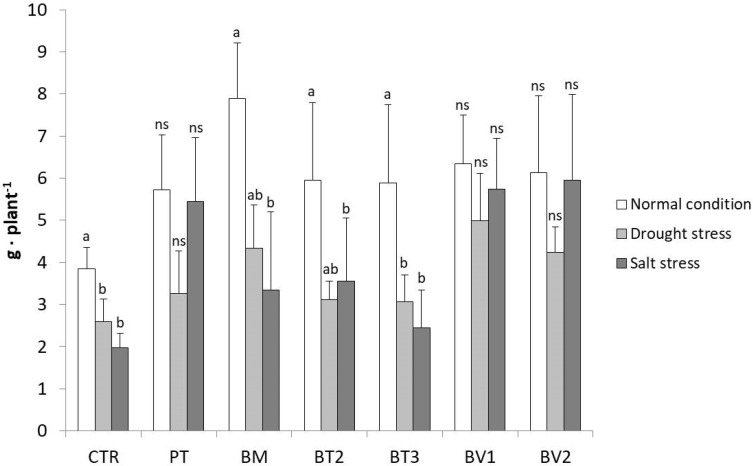
The graph shows the fresh root weights of the second greenhouse test compared between the three different conditions (normal, water stress, salt stress). Mean values (±SD); (^a, b^) different letters in each graphs represent statistically significant differences based on Tukey’s post hoc test (*p* ≤ 0.05), *ns* no significant effect. CTR: control thesis, PT: *P. taiwanensis* T_2_C, BM: *B. mojavensis* CSS_C9, BT2: *B. tropicus* PB_7, BT3: *B. tequilensis* CSS_7_3, BV1: *B. velezensis* PB_8, and BV2: *B. velezensis* CSS_12_1.

This cross-sectional comparison of the strains across different treatments is illustrated in Figure 7, focusing on the FW of the roots which showed the best effect among PGPR treatments. Notably, the results clearly highlighted the superior performance of the *Bacillus velezensis* strains (BV1 and BV2). Specifically, for BV2, no significant differences were observed in root fresh weight across the three cultivation conditions. Similarly, no significant differences were found between BV1, BV1_D, and BV1_S or between BV2, BV2_D, and BV2_S, as the root fresh weights were comparable, demonstrating a stable and consistent root development regardless of the applied stress. In addition to *Bacillus velezensis*, *Pseudomonas taiwanensis* also showed a positive effect on root fresh weight, particularly under salt stress, where root biomass remained comparable to that observed under normal conditions. Although not specifically tested, the enhancement in root development under water stress could also be ascribed to the bacterium’s ability to produce exopolysaccharides (EPSs) [54,55]. Therefore, inoculation with both *Bacillus velezensis* strains appears to improve the ability of lettuce plants to cope with water stress, not only by improving root development, but also by facilitating water retention in soil. Although PT exhibited a moderate reduction under drought stress, its performance remained superior to the inoculated control, confirming its role in stress mitigation.

This differential response may be related to strain-specific stress tolerance mechanisms, as ion homeostasis is known to be improved by *P. taiwanensis* T_2_C [66] and salt-induced toxicity reduced, rather than water retention in the rhizosphere being directly promoted by this strain.

Under salt stress conditions, the enhancement of lettuce root development observed in the presence of *B. velezensis* and *P. taiwanensis* T_2_C highlights their multifaceted role in stress mitigation, with both strains effectively reducing stress-induced root growth inhibition. In particular, *B. velezensis* (PB_8 and CSS_12_1) and *P. taiwanensis* T_2_C appear to play a key role in maintaining root biomass under saline conditions, contributing to improved plant performance. By promoting the production of growth hormones and improving nutrient availability, *Bacillus velezensis* and *Pseudomonas taiwanensis* significantly boosted the resilience of lettuce plants to abiotic stressors.

Although the obtained results demonstrate clear mechanisms under controlled conditions, the low pot volume and the absence of interactions with the soil microbiome may overestimate the efficacy of the strain inoculation. Furthermore, the 33-day growth cycle does not evaluate the persistence of the inoculant in the long term. These findings should be validated in multi-location field trials with formulated inoculants.

## 4. Conclusions

This study successfully characterised several Plant Growth-Promoting Rhizobacteria (PGPR) strains, highlighting their potential to enhance plant growth and maintain their performance under stressful conditions. The in vitro tests confirmed that the mechanisms enhancing plant growth include phytohormone production, phosphate solubilisation, and siderophore production. The results of the greenhouse trials demonstrated that inoculation with the selected PGPR strains significantly improved plant growth parameters, such as root biomass, chlorophyll content, and photosynthetic activity, particularly under stress conditions. Among the strains tested, *Bacillus velezensis* PB_8, *Bacillus velezensis* CSS_12_1, and *Pseudomonas taiwanensis* T_2_C showed the most promising results in enhancing plant resilience to abiotic stresses, improving growth and quality parameters even in environments with high salinity and limited water availability. These findings suggest that the selected PGPR can be an effective, low-impact strategy for mitigating the adverse effects of water and salt stress in crops. The application of PGPR in sustainable agricultural practices could offer an alternative to chemical fertilisers and help improve crop productivity and quality in regions with challenging environmental conditions. Further studies are needed to better understand the mechanisms behind PGPR-mediated stress tolerance for improving crop quality and to optimise inoculant formulations and application in field conditions for long-term agricultural sustainability.

## Figures and Tables

**Table 1:** Summary of the strains and conditions in the second pot test in greenhouse, under normal cultivation (no stress), salt stress and water stress.

Strain	No Stress	Salt Stress	Drought Stress
CTR	CTR	CTR_S	CTR_D
***Pseudomonas taiwanensis*** ** T_2_C**	PT	PT_S	PT_D
***Bacillus mojavensis*** ** CSS_C9**	BM	BM_S	BM_D
***Bacillus velezensis*** ** PB_8**	BV1	BV1_S	BV1_D
***Bacillus tropicus*** ** PB_7**	BT1	BT1_S	BT1_D
***Bacillus tequilensis*** ** CSS_7_3**	BT2	BT2_S	BT2_D
***Bacillus velezensis*** ** CSS_12_1**	BV2	BV2_S	BV2_D

**Table 2:** Identification of the best-matching phylotypes of purified PCR products derived from the 16S rRNA amplification of the isolated strains.

Strain	Closet Match	Source	Accession Number *
T_2_C	*Pseudomonas taiwanensis*	*Zingiber officinale*	PQ656521
CSS_C9	*Bacillus mojavensis*	Coffee silver skin	MZ357958
CM_4_4	*Stenotrophomonas maltophilia*	Chicken manure	PQ656523
CSS_12_6	*Bacillus halotolerans*	Coffee silver skin	OP390833
T_1_R	*Pseudomonas plecoglossicida*	*Zingiber officinale*	PQ656522
PB_8	*Bacillus velezensis*	*Prunus persica*	PQ656524
QR_6	*Bacillus toyonensis*	*Quercus robur*	PQ656526
PB_7	*Bacillus tropicus*	*Prunus persica*	PQ656525
CSS_7_3	*Bacillus tequilensis*	Coffee silver skin	OP390829
CSS_12_1	*Bacillus velezensis*	Coffee silver skin	OP390823

* Provided by GeneBank (https://www.ncbi.nlm.nih.gov/genbank/) accessed on 8 June 2021.

**Table 3:** Results of in vitro tests on the characterisation of phosphate solubilisation, siderophore production and insol-3-acetic acid production after 24 h, 48 h and 72 h.

Strains	Phosphate Solubilisation	Siderophore Production	IAA (μg/mL) 24 h	IAA (μg/mL) 48 h	IAA (μg/mL) 72 h
*P. plecoglossicida* T_1_R	++	++	14.37 ± 0.17 ^c^	15.54 ± 0.28 ^g^	16.40 ± 0.32 ^g^
*P. taiwanensis* T_2_C	+++	++	14.71 ± 0.31 ^c^	18.41 ± 0.26 ^e^	27.28 ± 0.04 ^f^
*B. moyavensis* CSS_C9	+	++	14.24 ± 0.07 ^c^	28.44 ± 0.18 ^b^	41.48 ± 0.22 ^e^
*B. tropicus* PB_7	+	++	17.62 ± 0.21 ^b^	17.52 ± 0.19 ^f^	42.41 ± 0.06 ^d^
*B. tequilensis* CSS_7_3	++	++	38.61 ± 0.23 ^a^	42.56 ± 0.43 ^a^	54.48 ± 0.33 ^a^
*B. toyonensis* QR_6	-	-	4.14 ± 0.03 ^d^	9.31 ± 0.07 ^h^	9.64 ± 0.10 ^i^
*B. velezensis* PB_8	++	+	18.47 ± 0.26 ^b^	19.64 ± 0.29 ^d^	44.60 ± 0.25 ^c^
*B. velezensis* CSS_12_1	++	++	18.48 ± 0.04 ^b^	22.61 ± 0.26 ^c^	53.58 ± 0.21 ^b^
*B. halotolerans* CSS_12_6	+	++	19.51 ± 0.16 ^b^	21.34 ± 0.23 ^c^	36.42 ± 0.22 ^e^
*S. maltophilia* CM_4_4	+	++	14.22 ± 0.11 ^c^	15.54 ± 0.28 ^g^	15.74 ± 0.11 ^h^
s			**	***	***

The symbols indicate the following: -, no halos; +, small halos < 0.5 cm; ++, medium halos > 0.5 cm; +++, large halos > 1.0 cm; mean values (±SD); (^a, b, c, d, e, f, g, h, i^) different letters in each column represent statistically significant differences in indol-3-acetic acid production based on Tukey’s post hoc test (*p* ≤ 0.05). **: significant at *p* ≤ 0.01, ***: significant at *p* ≤ 0.001.

**Table 4:** Leaves fresh weight (FW), dry weight (DW), dry matter (DM%) in plants with optimal growth conditions in the preliminary experiment.

Experimental Theses	FW (g ∙ Plant^−1^)	DW (g) (g ∙ Plant^−1^)	DM (%)
CTR	126.59 ± 10.71	7.09 ± 0.68	5.61 ± 0.36
PP	121.12 ± 8.98	6.74 ± 0.31	5.59 ± 0.45
PT	125.35 ± 6.44	6.76 ± 0.62	5.39 ± 0.34
SM	123.94 ± 13.16	6.76 ± 0.78	5.47 ± 0.44
BH	125.23 ± 14.33	6.66 ± 0.74	5.33 ± 0.38
BM	121.64 ± 19.06	6.75 ± 1.06	5.55 ± 0.21
BT1	120.28 ± 13.47	6.42 ± 0.64	5.35 ± 0.32
BT2	127.63 ± 13.54	7.16 ± 0.63	5.62 ± 0.23
BT3	125.43 ± 14.72	6.99 ± 0.82	5.58 ± 0.35
BV1	126.60 ± 9.47	7.16 ± 0.55	5.66 ± 0.25
BV2	115.89 ± 9.40	6.26 ± 0.78	5.39 ± 0.39
*Significance*	*ns*	*ns*	*ns*

Mean values (±SD); *ns* effect not significant. CTR: control thesis, PP: *P. plecoglossicida* T_1_R, PT: *P. taiwanensis* T_2_C, SM: *S. maltophilia* CM_4_4, BH: *B. halotolerans* CSS_12_6, BM: *B. mojavensis* CSS_C9, BT1: *B. toyonensis* QR_6, BT2: *B. tropicus* PB_7, BT3: *B. tequilensis* CSS_7_3, BV1: *B. velezensis* PB_8, and BV2: *B. velezensis* CSS_12_1.

**Table 5:** Leaves chlorophyll a (Chlor a), chlorophyll b (Chlor b) content, and carotenoids (Car) content in plants in the preliminary experiment.

Experimental Theses	Chlor a (µg·g^−1^)	Chlor b (µg·g^−1^)	Car (µg·g^−1^)
CTR	38.38 ± 0.97 ^f^	15.94 ± 0.65 ^ef^	3851.29 ± 36.62 ^f^
PP	37.06 ± 0.58 ^f^	13.36 ± 4.44 ^f^	3223.42 ± 14.04 ^g^
PT	65.04 ± 0.41 ^a^	35.42 ± 0.88 ^a^	5386.09 ± 164.29 ^a^
SM	47.42 ± 0.6 ^d^	20.54 ± 0.27 ^cd^	4123.95 ± 10.09 ^cd^
BH	50.90 ± 0.31 ^c^	26.70 ± 0.53 ^b^	4289.48 ± 28.39 ^b^
BM	52.67 ± 0.70 ^b^	25.66 ± 0.93 ^b^	4276.42 ± 37.33 ^bc^
BT1	23.10 ± 0.09 ^h^	18.34 ± 1.20 ^de^	2701.09 ± 18.21 ^h^
BT2	31.50 ± 0.20 ^g^	20.14 ± 0.38 ^cde^	3340.21 ± 26.86 ^g^
BT3	43.03 ± 0.23 ^e^	22.76 ± 0.39 ^bc^	4023.15 ± 20.71 ^de^
BV1	42.53 ± 0.17 ^e^	21.16 ± 0.27 ^cd^	4286.38 ± 10.60 ^b^
BV2	42.24 ± 0.15 ^e^	20.61 ± 0.82 ^cd^	3948.91 ± 26.61 ^ef^
*Significance*	***	***	***

Mean values (±SD). (^a, b, c, d, e, f, g, h^) Different letters in each column represent statistically significant differences based on Tukey’s post hoc test (*p* ≤ 0.05). ***: significant at *p* ≤ 0.001. CTR: control thesis, PP: *P. plecoglossicida* T_1_R, PT: *P. taiwanensis* T_2_C, SM: *S. maltophilia* CM_4_4, BH: *B. halotolerans* CSS_12_6, BM: *B. mojavensis* CSS_C9, BT1: *B. toyonensis* QR_6, BT2: *B. tropicus* PB_7, BT3: *B. tequilensis* CSS_7_3, BV1: *B. velezensis* PB_8, and BV2: *B. velezensis* CSS_12_1.

**Table 6:** Roots fresh weight (FW), dry weight (DW), and dry matter (DM%) in plants in the preliminary experiment.

Experimental Theses	FW (g ∙ Plant^−1^)	DW (g ∙ Plant^−1^)	DM (%)
CTR	2.61 ± 1.31 ^bcd^	0.29 ± 0.14	11.22 ± 1.94 ^c^
PP	3.89 ± 0.88 ^ab^	0.65 ± 0.75	16.01 ± 5.95 ^c^
PT	5.32 ± 1.59 ^a^	0.47 ± 0.11	9.10 ± 2.07 ^c^
SM	1.30 ± 0.48 ^d^	0.34 ± 0.09	28.33 ± 8.77 ^a^
BH	1.76 ± 0.5 ^cd^	0.38 ± 0.05	22.50 ± 4.63 ^b^
BM	4.95 ± 1.69 ^a^	0.51 ± 0.21	10.56 ± 3.22 ^c^
BT1	3.63 ± 1.01 ^abc^	0.42 ± 0.07	11.97 ± 2.28 ^c^
BT2	5.19 ± 1.26 ^a^	0.43 ± 0.04	8.61 ± 1.92 ^c^
BT3	5.31 ± 1.87 ^a^	0.43 ± 0.12	8.47 ± 1.95 ^c^
BV1	3.65 ± 0.81 ^abc^	0.39 ± 0.07	10.93 ± 1.98 ^c^
BV2	5.62 ± 1.91 ^a^	0.46 ± 0.12	8.57 ± 1.97 ^c^
*Significance*	***	*ns*	*

Mean values (±SD). (^a, b, c, d^) Different letters in each column represent statistically significant differences based on Tukey’s post hoc test (*p* ≤ 0.05).*: significant at *p* ≤ 0.05; ***: significant at *p* ≤ 0.001; *ns*: no significant effect. CTR: control thesis, PP: *P. plecoglossicida* T_1_R, PT: *P. taiwanensis* T_2_C, SM: *S. maltophilia* CM_4_4, BH: *B. halotolerans* CSS_12_6, BM: *B. mojavensis* CSS_C9, BT1: *B. toyonensis* QR_6, BT2: *B. tropicus* PB_7, BT3: *B. tequilensis* CSS_7_3, BV1: *B. velezensis* PB_8, and BV2: *B. velezensis* CSS_12_1.

**Table 7:** Leaves fresh weight (FW), dry weight (DW), dry matter (DM%), chlorophyll a (Chlor a), chlorophyll b (Chlor b) content, and carotenoids (Car) content in plants without abiotic stresses.

Experimental Theses	FW (g ∙ Plant^−1^)	DW (g ∙ Plant^−1^)	DM (%)	Chlor a (µg·g^−1^)	Chlor b (µg·g^−1^)	Car (µg·g^−1^)
CTR	97.54 ± 7.44 ^b^	3.92 ± 0.31	4.03 ± 0.27	77.93 ± 0.37 ^f^	44.36 ± 0.69 ^e^	8049.96 ± 24.92 ^f^
PT	96.56 ± 19.91 ^b^	3.56 ± 1.05	3.77 ± 1.03	169.08 ± 0.27 ^b^	96.30 ± 0.47 ^c^	17,671.3 ± 23.59 ^d^
BM	116.31 ± 6.19 ^a^	4.30 ± 0.27	3.70 ± 0.26	164.16 ± 0.29 ^c^	91.58 ± 0.24 ^d^	17,702.3 ± 18.91 ^cd^
BT2	107.98 ± 3.08 ^ab^	4.08 ± 0.14	3.78 ± 0.15	156.30 ± 0.34 ^e^	142.65 ± 2.91 ^f^	32,954.51 ± 94.32 ^e^
BT3	111.95 ± 10.60 ^ab^	4.35 ± 0.47	3.89 ± 0.28	191.94 ± 0.25 ^a^	106.31 ± 0.28 ^a^	21,640.51 ± 14.17 ^a^
BV1	110.54 ± 6.14 ^ab^	4.11 ± 0.21	3.73 ± 0.23	191.33 ± 0.38 ^a^	104.42 ± 0.42 ^b^	17,780.71 ± 14.09 ^c^
BV2	109.89 ± 8.65 ^ab^	4.18 ± 0.37	3.81 ± 0.35	158.81 ± 0.17 ^d^	97.78 ± 0.38 ^c^	19,693 ± 23.61 ^b^
*Significance*	*	*ns*	*ns*	***	***	***

Mean values (±SD). (^a, b, c, d, e, f^) Different letters in each column represent statistically significant differences based on Tukey’s post hoc test (*p* ≤ 0.05). * *p* ≤ 0.05, *** *p* ≤ 0.001, *ns* no significant effect. CTR: control thesis, PT: *P. taiwanensis* T_2_C, BM: *B. mojavensis* CSS_C9, BT2: *B. tropicus* PB_7, BT3: *B. tequilensis* CSS_7_3, BV1: *B. velezensis* PB_8, and BV2: *B. velezensis* CSS_12_1.

**Table 8:** Leaves fresh weight (FW), dry weight (DW), dry matter (DM%), chlorophyll a (Chlor a), chlorophyll b (Chlor b) content, and carotenoids (Car) content in plants with saline stress.

Experimental Theses	FW (g ∙ Plant^−1^)	DW (g ∙ Plant^−1^)	DM (%)	Chlor a (µg·g^−1^)	Chlor b (µg·g^−1^)	Car (µg·g^−1^)
CTR_S	49.48 ± 3.99 ^ab^	3.19 ± 0.36	6.44 ± 0.46	79.37 ± 0.23 ^f^	48.59 ± 0.45 ^d^	8036.31 ± 93.60 ^f^
PT_S	52.54 ± 2.68 ^ab^	2.99 ± 0.17	5.70 ± 0.32	174.67 ± 1.12 ^a^	91.29 ± 0.17 ^b^	16,757.31 ± 4.53 ^b^
BM_S	49.07 ± 7.31 ^ab^	3.12 ± 0.51	6.41 ± 1.15	104.27 ± 0.14 ^e^	85.89 ± 0.12 ^c^	16,425.81 ± 18.886 ^bc^
BT2_S	53.84 ± 4.93 ^ab^	3.06 ± 0.33	5.70 ± 0.71	78.40 ± 0.14 ^f^	45.46 ± 0.12 ^d^	8945.13 ± 9.39 ^e^
BV3_S	54.61 ± 6.22 ^ab^	2.93 ± 0.27	5.38 ± 0.22	172.54 ± 0.31 ^b^	105.46 ± 0.30 ^a^	21,723.71 ± 23.60 ^a^
BV1_S	58.61 ± 6.22 ^a^	3.21 ± 0.27	5.50 ± 0.35	143.76 ± 0.10 ^d^	82.98 ± 0.04 ^c^	15,539.41 ± 23.72 ^d^
BV2_S	43.53 ± 15.49 ^b^	2.63 ± 0.81	6.17 ± 0.45	151.15 ± 0.53 ^c^	90.51 ± 2.94 ^b^	16,287.91 ± 14.17 ^c^
*Significance*	*	*ns*	*ns*	***	***	***

Mean values (±SD); (^a, b, c, d, e, f^) different letters in each column represent statistically significant differences based on Tukey’s post hoc test (*p* ≤ 0.05). * *p* ≤ 0.05, *** *p* ≤ 0.001, *ns* no significant effect. CTR_S: control thesis, PT_S: *P. taiwanensis* T_2_C, BM_S: *B. mojavensis* CSS_C9, BT2_S: *B. tropicus* PB_7, BT3_S: *B. tequilensis* CSS_7_3, BV1_S: *B. velezensis* PB_8, and BV2_S: *B. velezensis* CSS_12_1.

**Table 9:** Leaves fresh weight (FW), dry weight (DW), dry matter (DM%), chlorophyll a (Chlor a), chlorophyll b (Chlor b) content, and carotenoids (Car) content in plants with water stress.

Experimental Theses	FW (g ∙ Plant^−1^)	DW (g ∙ Plant^−1^)	DM (%)	Chlor a (µg·g^−1^)	Chlor b (µg·g^−1^)	Car (µg·g^−1^)
CTR_D	51.30 ± 13.08 ^a^	2.42 ± 0.35	4.84 ± 0.66 ^ab^	99.66 ± 0.09 ^e^	45.79 ± 0.14 ^c^	8979.89 ± 15.61 ^f^
PT_D	36.57 ± 6.32 ^b^	2.08 ± 0.14	5.78 ± 0.67 ^a^	237.33 ± 0.22 ^b^	125.01 ± 0.45 ^b^	28,855.5 ± 18.87 ^d^
BM_D	44.27 ± 7.50 ^ab^	2.20 ± 0.35	4.98 ± 0.31 ^ab^	244.87 ± 0.82 ^a^	146.80 ± 3.05 ^a^	30,453.41 ± 141.42 ^b^
BT2_D	37.23 ± 4.23 ^b^	2.13 ± 0.48	5.69 ± 0.91 ^ab^	239.19 ± 0.47 ^b^	142.65 ± 2.91 ^a^	32,954.51 ± 94.33 ^a^
BV3_D	42.46 ± 4.95 ^ab^	2.34 ± 0.19	5.56 ± 0.69 ^ab^	211.97 ± 0.17 ^d^	121.61 ± 0.38 ^b^	27,793.2 ± 141.37 ^e^
BV1_D	44.23 ± 6.22 ^ab^	2.05 ± 0.19	4.67 ± 0.35 ^b^	225.58 ± 1.64 ^c^	127.47 ± 0.21 ^b^	28,957.4 ± 94.01 ^d^
BV2_D	44.39 ± 7.10 ^ab^	2.31 ± 0.28	5.23 ± 0.39 ^ab^	237.32 ± 0.31 ^b^	127.58 ± 0.31 ^b^	29,332.1 ± 14.18 ^c^
*Significance*	*	*ns*	*	***	***	***

Mean values (±SD). (^a, b, c, d, e, f^) Different letters in each column represent statistically significant differences based on Tukey’s post hoc test (*p* ≤ 0.05). * *p* ≤ 0.05, *** *p* ≤ 0.001, *ns* no significant effect. CTR_D: control thesis, PT_D: *P. taiwanensis* T_2_C, BM_D: *B. mojavensis* CSS_C9, BT2_D: *B. tropicus* PB_7, BT3_D: *B. tequilensis* CSS_7_3, BV1_D: *B. velezensis* PB_8, and BV2_D: *B. velezensis* CSS_12_1.

## Data Availability

The raw data supporting the conclusions of this article will be made available by the authors on request.
